# Lightweight ViT Model for Micro-Expression Recognition Enhanced by Transfer Learning

**DOI:** 10.3389/fnbot.2022.922761

**Published:** 2022-06-30

**Authors:** Yanju Liu, Yange Li, Xinhai Yi, Zuojin Hu, Huiyu Zhang, Yanzhong Liu

**Affiliations:** ^1^School of Mathematics and Information Science, Nanjing Normal University of Special Education, Nanjing, China; ^2^School of Computer and Control Engineering, Qiqihar University, Qiqihar, China

**Keywords:** computer vision, deep learning, convolutional neural network, vision transformer, micro-expression recognition

## Abstract

As opposed to macro-expressions, micro-expressions are subtle and not easily detectable emotional expressions, often containing rich information about mental activities. The practical recognition of micro-expressions is essential in interrogation and healthcare. Neural networks are currently one of the most common approaches to micro-expression recognition. Still, neural networks often increase their complexity when improving accuracy, and overly large neural networks require extremely high hardware requirements for running equipment. In recent years, vision transformers based on self-attentive mechanisms have achieved accuracy in image recognition and classification that is no less than that of neural networks. Still, the drawback is that without the image-specific biases inherent to neural networks, the cost of improving accuracy is an exponential increase in the number of parameters. This approach describes training a facial expression feature extractor by transfer learning and then fine-tuning and optimizing the MobileViT model to perform the micro-expression recognition task. First, the CASME II, SAMM, and SMIC datasets are combined into a compound dataset, and macro-expression samples are extracted from the three macro-expression datasets. Each macro-expression sample and micro-expression sample are pre-processed identically to make them similar. Second, the macro-expression samples were used to train the MobileNetV2 block in MobileViT as a facial expression feature extractor and to save the weights when the accuracy was highest. Finally, some of the hyperparameters of the MobileViT model are determined by grid search and then fed into the micro-expression samples for training. The samples are classified using an SVM classifier. In the experiments, the proposed method obtained an accuracy of 84.27%, and the time to process individual samples was only 35.4 ms. Comparative experiments show that the proposed method is comparable to state-of-the-art methods in terms of accuracy while improving recognition efficiency.

## Introduction

In everyday social communication, facial expressions provide a wealth of emotional information. Typically, facial macro-expressions last about 4–5 s and are easily perceived by humans. On the other hand, micro-expressions are rapid involuntary facial movements that reveal a person's true feelings. Facial micro-expression recognition has many applications and has been an active research area in recent years. Accurate micro-expression recognition is complicated due to their subtlety and transient nature. Humans can detect micro-expressions if a video with many frames is played in slow motion (Ekman, 2009). However, the accuracy of trained humans in recognizing micro-expressions in real-life scenarios is only 47% (Frank et al., 2009). Micro-expression recognition technology has received more attention due to its wide application in various fields, such as police interrogation, clinical diagnosis, depression analysis, lie detection, business negotiation, and teaching aid.

With the development of machine learning and deep learning, as well as the proposal of benchmark datasets [e.g., SMIC (Li et al., 2013), CASME II (Yan et al., 2014), and SAMM (Davison et al., 2016)], research on micro-expression recognition has increased in recent years, and the methods used have gradually shifted from the initial manual extraction of features to deep learning to extract features for learning and training automatically. For example, in the early days of research, some researchers attempted to extract subtle changes in facial skin texture in micro-expressions using local binary patterns on three orthogonal planes (LBP-TOP) (Pfister et al., 2011; Davison et al., 2014) that better describe the dynamic texture and improved methods based on them (e.g., Ruiz-Hernandez and Pietikäinen, 2013; Wang et al., 2014; Huang et al., 2015, 2017). Another group of researchers sought to extract features of facial light changes when subjects made micro-expressions, such as directional mean optical flow (MDMO) (Liu et al., 2015), bi-weighted directional optical flow (Bi-WOOF) (Liong et al., 2018), and fuzzy optical flow directional histogram (FHOFO) (Happy and Routray, 2017). After extracting the features, the samples are classified by constructing classifiers. The more commonly used ones are support vector machines (Pfister et al., 2011; Huang et al., 2015; Liong et al., 2018) and random forest (Pfister et al., 2011; Davison et al., 2014). These methods improve the recognition of micro-expressions to a certain extent but still have difficulty in capturing too subtle facial changes. On the other hand, deep learning methods can automatically and efficiently extract features that are not easily detected and have become one of the mainstream methods for extracting features in recent years (Wang et al., 2018; Wu et al., 2020; Xia et al., 2020a). Therefore, deep learning methods are also used in micro-expression recognition for efficient feature extraction and classification capabilities. Examples range from pre-trained CNNs [e.g., OFF-ApexNet (Gan et al., 2019) and TSCNN (Song et al., 2019)] to combining CNNs with long- and short-term memory (Wang et al., 2018). After achieving high accuracy on single datasets, researchers have used cross-datasets and composite datasets on micro-expression recognition. The application scenarios for micro-expressions align with the cross-datasets and composite datasets. Therefore, the composite datasets were also used for the MEGC2019 dataset. However, the increase in accuracy is accompanied by an increase in the complexity of the network model. Fast and efficient processing is essential in specific scenarios, such as embedded systems for car driver monitoring or teaching aids for student comprehension recognition. These advantages often come at the cost of limited hardware resources. Therefore, in recent years, researchers have proposed lightweight convolutional neural networks such as ShuffleNetV2 (Ma et al., 2018) and MobileNetV3 (Howard et al., 2019) to improve efficiency, reduce the cost of hardware resources, and achieve better accuracy in micro-expression recognition (Xu et al., 2021). By contrast, attention-based models, particularly visual transforms (ViTs) (Dosovitskiy et al., 2020), are an alternative to convolutional neural networks (CNNs) for learning visual features. Briefly, the original ViT divides an image into a string of consecutive non-overlapping image blocks and then uses multi-headed self-attention in the transformer (Vaswani et al., 2017) to learn features between blocks in the image phase. The trend in this type of model is to increase the number of parameters in the ViT network to improve accuracy while introducing the problem of increased latency. By contrast, many realistic computer vision tasks, such as object detection and facial recognition, mostly need to be run on the fly on devices with limited hardware resources. To improve efficiency, the ViT model for such computer vision tasks should be lightweight, but its performance is much worse than the equally lightweight CNN. Moreover, the ViT model lacks the inductive bias characteristic of CNN models, which consider information to be spatially local, and therefore does not have the ability to extract and analyze global information that they have.

In order to make the micro-expression recognition model have the advantages of both the lightweight CNN and ViT, this study chooses MobileViT (Mehta and Rastegari, 2021) proposed by Apple as the model, which combines the lightweight CNN model MobileNetV2 with ViT so that the model as a whole has the characteristics of both, and the model consists of two parts: the MobileNetV2 block and the MobileViT block. In order to address the insufficient number of samples in the micro-expression dataset, the macro-expression migration learning method is used to avoid the inadequate feature extraction ability of the model, as well as the overfitting problem. At the same time, we used the random spatial transformation method to balance the number of samples of different emotion categories when dealing with the problem of the unbalanced number of emotion samples. In the pre-processing part of the dataset, we applied the same treatment to macro- and micro-expressions to make them as similar as possible, including taking out the vertex frames of each expression sample, extracting the facial regions, and cropping them to a fixed size after grayscale processing. Finally, due to the slight differences between the micro-expression samples, a support vector machine, which is more capable of discovering the maximum differences between samples, is used to classify the samples.

Our main contributions are summarized as follows:

The MobileNetV2 block in the MobileViT model is trained as a facial expression feature extractor through migration learning of macro-expressions, which can effectively improve the recognition accuracy and robustness in micro-expression recognition training.

In view of the insufficient number of samples in the micro-expression dataset and the unbalanced number of emotion categories, the data augmentation method is adopted to balance the samples and add L2 regularization to the convolutional and fully connected layers in the MobileViT model to avoid overfitting.

A grid search is introduced to allow some hyperparameters to be set to the most appropriate values before formal training.

The proposed method is comparable to the state-of-the-art MER method through a large number of comparative experiments.

The rest of the article is organized as follows: Section Related Work is a summary review of relevant research in recent years. Section Micro-Expression Recognition Model Design explains the proposed model and preparatory work. Section Experiments provides experimental results and performance evaluation. Finally, Section Conclusion and Future Work concludes the article with conclusions and future work.

## Related Work

This section summarizes the more popular deep learning methods for recognizing micro-expressions today and some research on the application of ViT in image classification.

In recent years, many researchers have considered deep learning as one of the effective methods for learning visual features. Neural network methods in deep learning have been widely used in image processing, video analysis, and speech recognition. In the absence of manual feature extraction, end-to-end neural network models are able to perform classification and prediction by learning numerous high-dimensional features and datasets with insufficient amounts of data. The convolutional neural network, one of the most widely used deep learning methods, is currently the leading approach in many image-related fields, such as large-scale object recognition (Guo et al., 2016) and face recognition (Schroff et al., 2015). In the past few years, CNNs have been heavily modified in terms of the increasing number of layers and block design, with famous successors such as AlexNet (Krizhevsky et al., 2012), VGGNet (Simonyan and Zisserman, 2014), and GoogLeNet (Szegedy et al., 2015). Despite the different network structures, deep learning models all benefit from the ability to learn high-dimensional representations from large datasets.

Recently, several studies applying deep learning for micro-expression recognition have emerged. Researchers used CNNs to extract features from micro-expression videos and further applied classifiers such as SVM to obtain classification results. Subsequently, Peng et al. (2017) designed the first end-to-end medium-scale neural network, called dual time-scale convolutional neural network (DTSCNN), for micro-expression recognition. The DTSCNN has two temporal channels and is designed for data with different temporal properties, for example, the cameras used for data collection have different frame rates. Each channel has only four convolutional layers and four pooling layers in order to avoid overfitting. The achieved recognition rate is about 10% higher than some previous state-of-the-art methods. Recently, Khor et al. (2018) proposed to train a network with convolutional and recursive layers for micro-expression recognition. Instead of using data enhancement on the dataset, they extracted optical flow features to enrich the input for each time step or specific time length. However, they did not achieve competitive results due to the occurrence of overfitting cases caused by using deep networks on small datasets. Moreover, for the nature of micro-expressions with a duration of <1/2 s, training the network on complete video clips may not be appropriate. Since the sample size of the micro-expression dataset is still very small, it cannot be adequately trained using deep convolutional neural networks, and the training cost is high, which is difficult for ordinary computer hardware resources to meet the training requirements. Xia et al. (2020b) analyzed the composite dataset, suggesting that a low-resolution and shallow network model would help improve the accuracy of the model trained on the composite dataset and proposed a recurrent convolutional network with a partially parameter-free module to validate their argument, showing that the proposed method outperforms state-of-the-art approaches. Another study by this author (Xia et al., 2018) proposed an end-to-end framework consisting of recursive convolutional networks (RCNNs) to recognize micro-expressions. The RCNN was used to learn subtly changing features and to recognize micro-expressions. Song et al. (2019) proposed a three-stream convolutional neural network (TSCNN) for micro-expression recognition and designed a module with dynamic temporal flow, static spatial flow, and local spatial flow for the TSCNN to learn and integrate temporal, whole face region, and local face region cues in micro-expression videos, respectively, for micro-expression recognition. The results are also compared to those of state-of-the-art approaches in many studies. Temporal jitter was used to enrich the training samples to facilitate the learning process, and the effectiveness of the method was validated on three spontaneous micro-expression datasets. Therefore, another researcher later used lightweight convolutional neural networks to solve the micro-expression recognition problem and achieved better results. For example, Belaiche et al. (2020) performed two ways of optimization based on ResNet18 to reduce the depth of the network by reducing the residual layers and using more compact optical flow features as input. Their proposed network rivals the accuracy of the state-of-the-art methods at that time while significantly reducing the necessary memory space. Xu et al. (2021) performed the micro-expression recognition task based on optical flow features and using a modified MobileNetV2, which improved the training efficiency but did not outperform the more complex deep neural network models in terms of accuracy.

In the field of computer vision, to find the region of interest (ROI) in an image and highlight the performance at that location, some researchers have introduced the self-attention mechanism widely used in natural language processing. For micro-expression recognition, this mechanism can help the network focus on the crucial feature regions of the face and reduce the negative influence of irrelevant facial areas and backgrounds. The ViT introduced by Dosovitskiy et al. (2020) directly inherited the architecture of natural language processing, based on multi-headed self-attentiveness, and applied to raw image patches for classification. However, they concluded that ViT struggles to generalize when the amount of data is insufficient. The convolution-free visual transformer DeiT proposed by Touvron et al. (2021) achieved a maximum of 83.1% on ImageNet accuracy and introduced a data-efficient training procedure that reduces the amount of data required for training. Even so, ViT struggles to achieve high accuracy rates at lightweight.

## Micro-Expression Recognition Model Design

**Figure 1:**
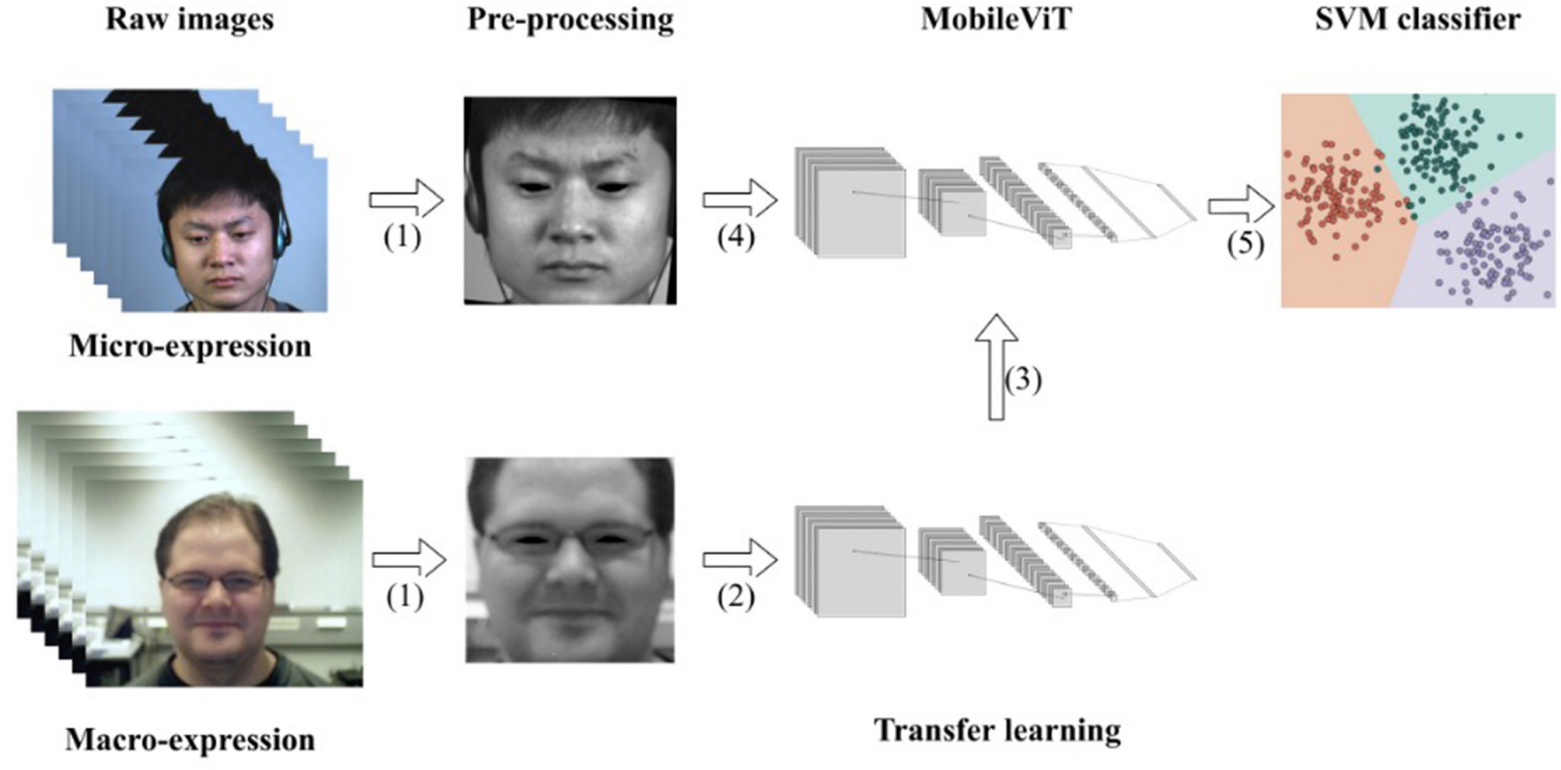
Flow chart of recognition model: (1) same pre-processing of the micro-expression image and macro-expression image sequences; (2) feeding macro-expressions into the network model for training and saving the weights when recognition accuracy is highest; (3) loading weights; (4) feeding micro-expression data into the model for training; (5) using the SVM classifier for classification. The micro-expression sample in the figure is from subject 1 of the CASME II dataset and the macro-expression sample is from subject 23 of the Oulu-CASIA NIR&VIS dataset.

Each part included in the model is described in detail in this section. The model consists of three main parts: pre-processing of macro- and micro-expressions, transfer learning of macro-expressions, and the MobileViT model. In pre-processing, to achieve better results in transfer learning, we used the same pre-processing approach for macro-expression and micro-expression samples. Their differences are minor after pre-processing. To solve the problem of the insufficient number of micro-expression samples, a composite dataset similar to MEGC2019 was used. Using transfer learning, the overfitting problem that tends to occur on small- and medium-sized datasets can be avoided to a certain extent. Through transfer learning, part of the convolutional layer of MobileViT can be trained as an extractor of facial expression features. Subsequent training will be effective in extracting subtle facial features. The MobileViT model has been partially adapted and improved for the micro-expression recognition task by adding L2 regularization to the convolutional and fully connected layers to avoid overfitting during training. Unlike micro-expressions, which have minimal variation between samples, the SVM classifier was chosen to find differences between samples. The flow chart of the recognition model is shown in Figure 1.

### Pre-processing

**Figure 2:**
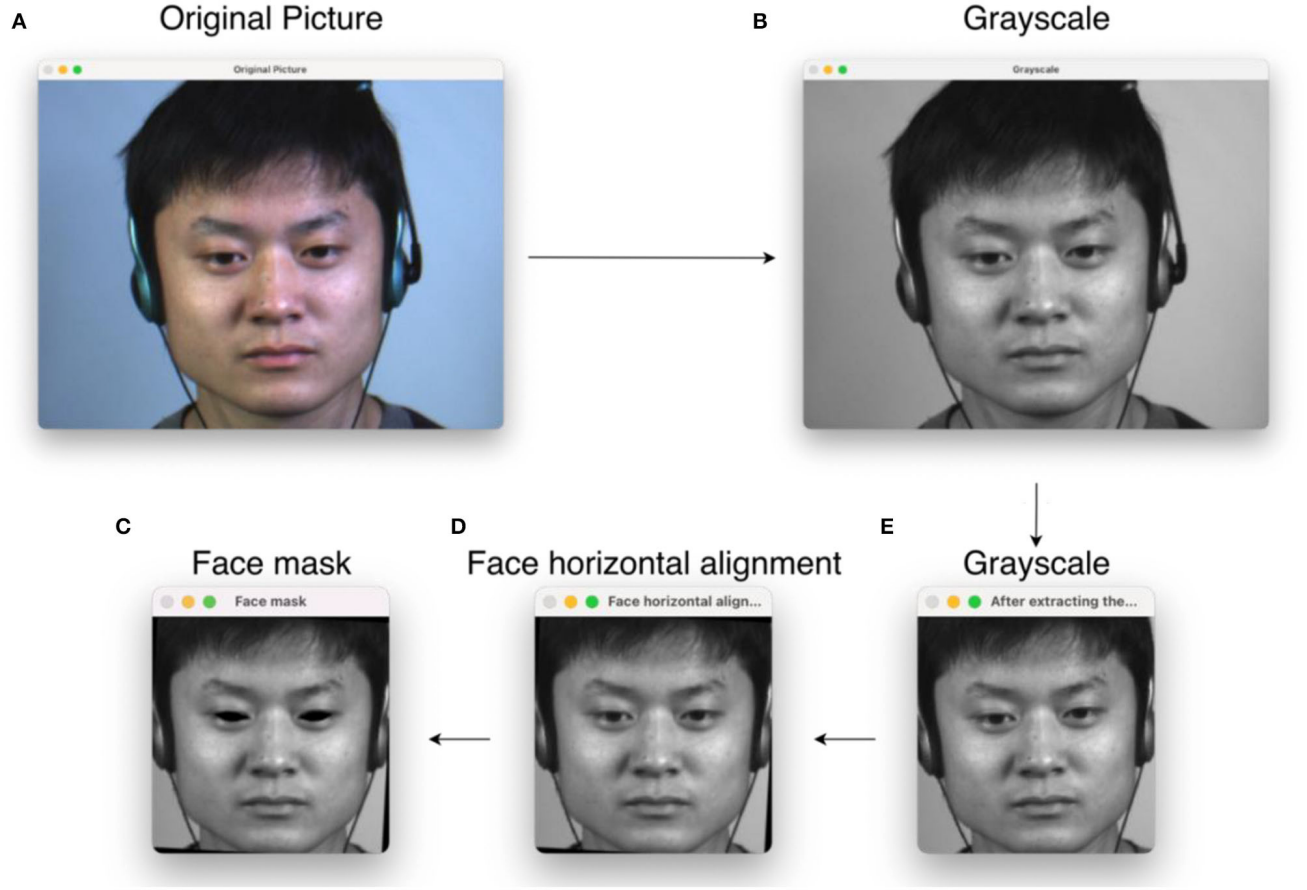
Pre-processing flow chart, where **(A)** original image; **(B)** processed to grayscale image; **(C)** extracted facial region and cropped size; **(D)** adjusted facial angle so that the face is horizontal; **(E)** masked in the eye. The micro-expression sample in the figure is from subject 1 of the CASME II dataset.

Usually, in image classification tasks, a pre-processing step is essential. Since datasets are produced with different sampling equipment and laboratory conditions, this will lead to differences in image resolution, brightness, and other metrics. When drawing samples from the macro-expression dataset, we need to pay attention to whether their backgrounds are close to those of micro-expression samples to avoid using noise as one of the features in migration learning. In this study, the original image sequence provided by the dataset is selected for pre-processing. The training cost can be significantly reduced by pre-processing, such as color processing and cropping size steps. In this study, the complete micro-expression image sequence is a continuous image including the start frame, apex frame, and the end frame, where the vertex frame is the image of the most prominent moment of micro-expression, so the vertex frame of each expression sample is first taken out and converted to grayscale image in the pre-processing step. Since the images are processed to learn features, rather than manipulating the images themselves, the color information is not essential in micro-expression recognition. After that, the facial region is first extracted using OpenCV and Dlib toolkit and modified in size to 256 × 256 pixels and marked with facial marker points. The coordinates of the left eye *A*(*x*_*L*_, *y*_*L*_) and the right eye *B*(*x*_*R*_, *y*_*R*_) are obtained from the marker points. The coordinates of the center of the two eyes *C*(*x*_*C*_, *y*_*C*_) are calculated from the coordinates of the two eyes, and the angle γ required to rotate the face to horizontal is calculated from the coordinates of the center and the coordinates of the left eye. Finally, the eyes are masked to avoid extraneous noise from eye movements. The pre-processing flow chart is shown in Figure 2. Its angle calculation equation is given as follows:


$$y=arctan\;\frac{y_{C}-\;y_{A}}{x_{C}-x_{A}}$$

The number of samples from different emotion categories is not close to each other in either the macro- or micro-expression dataset, so a data enhancement approach of random spatial transformations is introduced in processing. When pre-processing a small number of emotion samples from a particular emotion category, a small random horizontal or vertical shift of its pixel positions is performed, or the samples are subjected to a horizontal flip operation. In this way, the number of samples contained in each emotion class is effectively balanced, and the preference for a particular emotion class is reduced during training. After pretreatment, the samples are also carefully observed for facial integrity and horizontal facial posture to ensure that each sample meets the experimental requirements.

### Transfer Learning of Macro-Expressions

Some deep learning studies will train the network model on the ImageNet dataset (Russakovsky et al., 2015) and then fine-tune it before using it. By contrast, micro-expression recognition is different from other image recognition classification tasks in that all images are facial image data, so training on the ImageNet dataset is not appropriate. In this study, we choose to train MobileViT on four macro-expression datasets. They are the extended Cohn-Kanade dataset (CK+) (Lucey et al., 2010), Oulu-CASIA NIR&VIS facial expressions (Zhao et al., 2011), Jaffe (Lyons et al., 1998), and MUG Facial Expression (Aifanti et al., 2010). From the four macro-expression datasets, 9,342 images were selected for migration learning, including 3,085 samples of positive emotions, 3,263 samples of negative emotions, and 2,994 samples of surprising emotions. We take samples by looking at factors such as background and head tilt angle to get as close as possible to the micro-expression samples taken.

**Figure 3:**
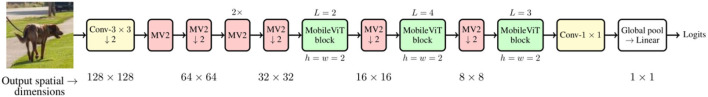
MobileViT architecture, Conv-*n* × *n* represents the standard n/times n convolution, MV2 refers to the MobileNetv2 block, and the block undergoing downsampling is marked with ↓2 (Mehta and Rastegari, 2021).

After pre-processing, they were fed into the MobileViT model for training. The overall architecture of MobileViT is given in Figure 3, and the detailed parameters of the model are listed in Table 1. The MobileViT model mainly includes MobileNetV2 block and MobileViT block, and the role of MobileNetV2 block is especially to downsample, extract local information, and extract local features. It is also the main module of transfer learning training, through the training of a high number of macro-expression samples, to get the excellent facial expression to feature extraction when the classification. The weights of the MobileNetV2 block are saved when the highest accuracy is achieved, and the MobileViT module contains standard convolution, point convolution, and a transformer, which is mainly used to extract global information so that the transformer includes the characteristics of convolution. Once the macro-expression samples are fed into the MobileViT model, first, the tensor is subjected to a layered 3 × 3 standard convolution and 2-fold downsampling to reduce the dimension of the tensor and increase the number of channels. Then four MobileNetV2 (Sandler et al., 2018) (MV2) blocks are input and are twice downsampled, and the two downsamplings are not adjacent to each other, using Swish (Elfwing et al., 2018) as the activation function, and the dimension of the tensor is 32 × 32. The tensor is fed into the MobileViT and MobileNetV2 blocks alternately, where the MobileViT block extracts the global information of the tensor and outputs it, as detailed in 3.3. The MobileNetV2 block continues to extract the local information and continues to double downsample. This process is carried out in two rounds to obtain a tensor of dimension 8 × 8, which is convolved with 1 × 1 and channel compressed. Finally, the result is output by the classifier after global average pooling.

**Table 1:** MobileViT architecture, where *d* represents the input size of the conversion layer in the MobileViT block.

**Layer**	**Output size**	**Output stride**	**Repeat**	**Output Channels**
Image	256 ×256	1		
Conv-3 ×3, ↓2 MV2	128 ×128	2	1 1	16 32
MV2, ↓2 MV2	64 ×64	4	1 2	64 64
MV2, ↓2 MobileViT block (*L* = 2)	32 ×32	8	1 1	96 96 (*d* = 144)
MV2, ↓2 MobileViT block (*L* = 4)	16 ×16	16	1 1	128 128 (*d* = 192)
MV2, ↓2 MobileViT block (*L* = 3) Conv-1 ×1	8 ×8	32	1 1 1	160 160 (*d* = 240) 640
Global pool Linear	1 ×1	256	1	1,000
Network Parameters			5.6 M

*By default, the kernel size n is set to 3 in the Mobile ViT block and the space size of the block (height h and width w) is set to 2*.

In addition, to improve the robustness of the network, pixel fine-tuning with probability 0.3 (maximum value 20) was performed on the images in the training set. A batch gradient descent method with a momentum of 0.9 was used during the training process. The batch size was set to 32, and the learning rate was initialized to 0.01. After 100 epochs, the average recognition rate for the three types of macro-expressions reached 98.45%. After a model has undergone migration learning, it needs to be fine-tuned to get higher accuracy in subsequent training. It is usually used to freeze the weights of all convolutional layers and gradually unfreeze the convolutional layers as the number of training iterations increases until all are unfroze and trained with the complete data.

### Optimized MobileViT Model

After the model has undergone migration learning, the micro-expression samples will be fed into the fine-tuned model for recognition and classification. The main reason lightweight CNNs are more efficient and have lower latency than conventional CNNs is the proposed separable convolution (Chollet, 2017). The separable convolution is general enough to replace the network model in most vision tasks. It is easy to train, effectively reducing the network model size and lowering the latency. However, its problem is that the separable convolution is still local in space, and it is challenging to extract global features. ViT, as transformer's attempt in computer vision concerns, has obtained better results in image classification problems in recent years. Similar to transformer's operation, a one-dimensional learnable position encoding is added to each patch to preserve its spatial information. The assembly is fed into the encoder as joint embeddings. ViT inserts a learnable category identity whose state at the output of the transformer's encoder is used as the performance classification criteria. In addition, a two-dimensional permutation method complements the pre-trained positional encoding to maintain a consistent order of the blocks when inputting images of arbitrary resolution so that global features can be obtained. However, it does not introduce an image-specific inductive bias and does not generalize well in the presence of insufficient training data.

**Figure 4:**
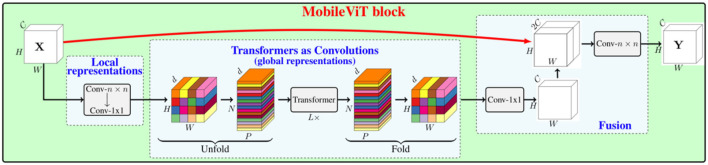
MobileViT block, where Conv-*n* × *n* represents the standard *n*×*n* convolution.

To solve the aforementioned problem, the MobileViT module is introduced, which aims to enable the model to acquire the samples' global features and add a CNN-specific inductive bias to ViT. As shown in Figure 4, first, a standard convolutional layer with *n*×*n* is applied for a given image input tensor **X**∈ℝ^*H*×*W*×*C*^ (where *H, W, and C* are the width, height, and number of channels of the image, respectively), to which a point convolutional layer is connected, yielding $X\in {\mathbb{R}}^{H\times W\times C}$. An *n*×*n* convolutional layer encodes local spatial information. By contrast, the point convolutional layer projects the tensor to the high-dimensional space by learning linear combinations of the input channels. After ensuring the effective perceptual field of having *H*×*W*, ViT with multi-headed self-attentiveness is chosen for the long-range non-local dependence. The method is to expand **X**_*L*_ into *N* non-overlapping flattened blocks $X_{U}\in {\mathbb{R}}^{P\times N\times d}$, where *P* = *wh*, $N=\frac{HW}{P}$ is the number of blocks, and *h* ≤ *n* and *w* ≤ *n* are the height and width of a block, respectively. For each *p*∈{1, ⋯ , *P*}, the relationship between the blocks is encoded by applying a transformer. $X_{G}\in {\mathbb{R}}^{P\times N\times d}$ is obtained as follows:


$${\text{X}}_{\text{G}}\left(\text{p}\right)=\text{Transforme r}\left({\text{X}}_{\text{U}}\left(p\right)\right),1\leq p\leq P$$

**Figure 5:**
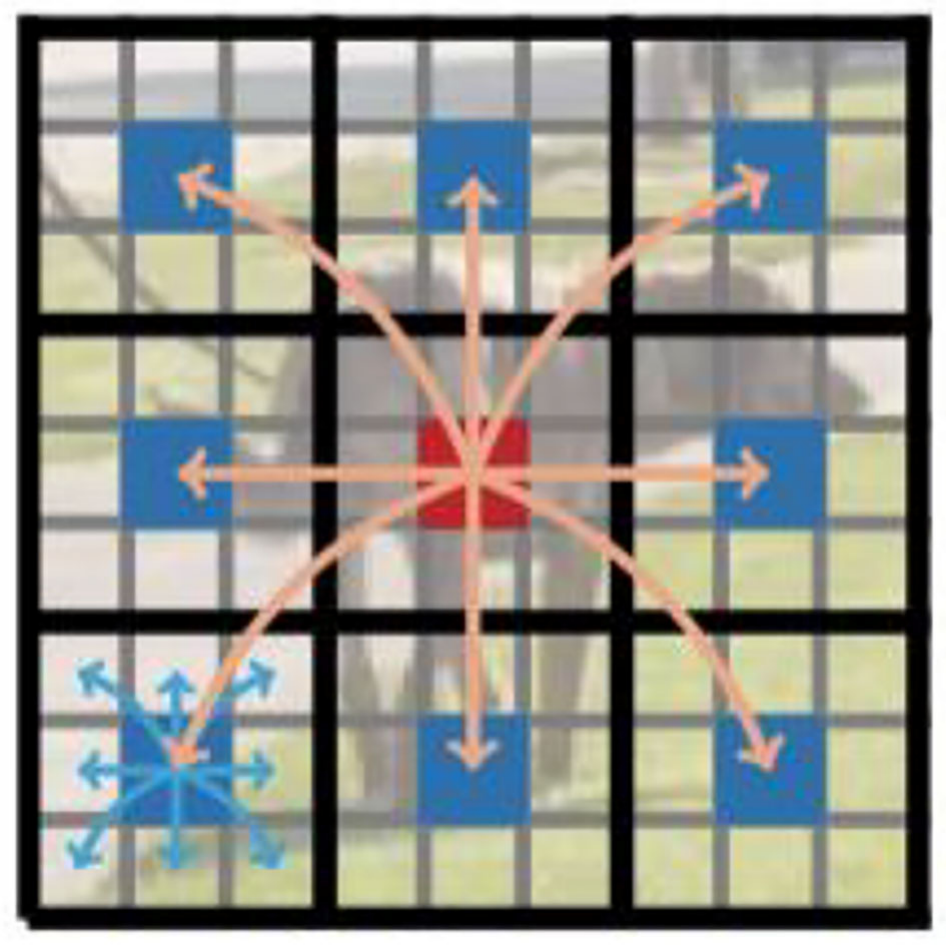
Each pixel sees every other pixel in the MobileViT block. In this example, the red pixel uses the transformer to focus on the blue pixel (the pixel in the corresponding position in the other blocks). Because the blue pixel already encodes the information of neighboring pixels using convolution, this allows the red pixel to encode the information of all pixels in the image. Here, each cell in the black and gray grids represents a block and a pixel, respectively.

Unlike ViT, which loses the spatial order of pixels, MobileViT loses neither the block order nor the spatial order of pixels within each block. Therefore, $X_{G}\in {\mathbb{R}}^{P\times N\times d}$ can be collapsed to obtain $X_{F}\in {\mathbb{R}}^{H\times W\times d}$. Then, **X**_*F*_ is projected to the low C-dimensional space using point-to-point convolution and combined with **X** by the join operation. Another *n*×*n* convolution layer is then used to fuse the local and global features in the tandem tensor and output. Since **X**_*U*_(*p*) uses convolution to encode local information in the *n*×*n* region and **X**_*G*_(*p*) encodes the global information of the P blocks at the pth position, each pixel in **X**_*G*_ can encode the information of all pixels in **X**, as shown in Figure 5. Thus, the MobileViT block has no loss of an effective sensory field.

The convolution kernel size *n* = 3 is used in the MobileViT block in this experiment, the spatial dimension of the feature map is usually a multiple of 2, and it is only *h, w* ≤ *n* when it ensures that no pixel information is lost, so *h* = *w* = 2 is set at all spatial levels. The converter layer in MobileViT requires a *d*-dimensional input, as shown in Figure 4, and the output dimension of the first feedforward layer in the transformer layer is set to the default value of 2*d*, instead of 4*d*, in the standard transformer block of Vaswani et al. (2017).

In the original ViT, for a given block, the spatial information is converted to subliminal by learning a linear combination of pixels. The global information is then encoded by learning inter-block information using a transformer. Thus, these models lose the image-specific inductive bias, which is inherent to CNNs. So they need deeper models to learn visual representations. By contrast, MobileViT uses a combination of convolution and transformer to give MobileViT blocks convolution-like properties while allowing global processing. This modeling capability enables the design of shallow and narrow lightweight network models.

The micro-expression dataset is characterized by a small sample size and very subtle differences between samples. In order to make the MobileViT model better cope with the micro-expression recognition problem, first, the weight decay rate of the model is adjusted through several experiments, gradually increasing from 0.0001 to 0.1, and finally, the model accuracy is highest at 0.01. Second, in the selection of the classifier, the SVM classifier can better find the difference between samples and thus improve the classification accuracy.

## Experiments

This section evaluates the proposed approach with several popular macro-expression datasets and three benchmark micro-expression datasets. Information about the datasets is first provided, followed by the experimental details. Finally, the results of some related works are compared and analyzed.

### Datasets

The quality of the images in the dataset significantly impacts the experimental results, so the more popular macro-expression and micro-expression datasets were chosen for this study. The datasets used in the experiments are all public datasets, obtained by request through the dataset manager. The number of emotion categories in the experiment was 3: positive emotions, negative emotions, and surprise. Positive emotions included only happiness, and negative emotions contained anger, disgust, and sadness. The surprise category of emotions, on the other hand, needed to be categorized separately as there was not enough information to determine their causes. The macro-emotion dataset for transfer learning and the micro-emotion dataset for core experiments are described as follows.

#### Macro-Expression Dataset Datasets

The CK+ dataset consists of 593 video clips from 123 subjects. Motion units were labeled on the last frame of each image sequence. Among the 593 clips, 327 had emotion type labels, namely, anger, contempt, disgust, fear, happiness, sadness, and surprise. Each clip starts from the normal facial expression frame and ends at the vertex frame. The last three frames were selected from the corresponding video clips of the three types of emotions desired in the experiment.

The Oulu-CASIA NIR&VIS facial expression dataset contains 80 subjects aged between 23 and 58 years and includes six typical categories of expressions, namely, happiness, sadness, surprise, anger, fear, and disgust. The image resolution was 320 × 240 pixels. All of these videos had two imaging systems captured, namely, the near-infrared and visible systems. The last three frames of each video captured by the visible light system were selected in this experiment.

Jaffe was published in 1998. The database contains 219 images of seven facial expressions (six basic facial expressions + one neutral expression) posed by 10 Japanese female models. Each woman was asked to perform seven types of expressions, namely, sad, happy, angry, disgusted, surprised, fearful, and neutral. In this experiment, the image with the correct expression label was selected from the corresponding images.

The MUGFE database consists of 1,032 video clips from 86 subjects, with 35 female and 51 male individuals, all of them being Caucasians between 20 and 35 years of age. The frame rate was 19 fps, and the resolution was 896/times 896 pixels. One part of the database contained six basic expressions, and the other part included laboratory-induced expressions. Each clip contained between 50 and 160 frames, starting and ending with the normal expression frame and ending with the vertex frame in between; six to 10 frames close to the vertex were selected from each segment in this experiment.

In the four aforementioned macro-expression datasets, 9,342 images were selected for migration learning, including 3,085 samples of positive emotions, 3,263 samples of negative emotions, and 2,994 samples of surprising emotions.

#### Micro-Expression Datasets

The SMIC dataset contains 164 micro-expression clips from 16 subjects, six females and ten males. The collection was done by cameras with different frame rates. These subjects underwent emotion to capture in an interrogation room environment, and the experiment also contained punishment and threat mechanisms that suppressed irrelevant facial expressions of the participants.

The CASMEII dataset contains 247 micro-expression clips from 26 subjects, captured using a camera with a frame rate of 200 fps, producing more images in the same amount of time and at a higher resolution than SMIC.

The SAMM dataset contains 159 micro-expressions from 32 subjects, achieving gender balance while including multiple ethnicities and nationalities, with a resolution of 400 × 400 in the facial region, the highest of any dataset. The acquisition process uses a variety of LED devices to ensure the stability of the light.

In the CASMEII and SAMM datasets, the vertex frames were labeled, while the SMIC dataset did not label the vertex frames, so the middle image of the expression image sequence was selected as the vertex frame in the SMIC dataset. Finally, 216 positive emotion samples, 231 negative emotion samples, and 170 surprised emotion samples were selected from the aforementioned three micro-expression datasets.

### Experimental Setup

For the experimental setup, the authors of MobileViT provided three scales of models, but due to the high complexity of the micro-expression recognition task, we chose the largest scale model. The hardware platform on which the models were run was an Intel Core i99980HK CPU, a 32 GB RAM, and an AMD Radeon Pro 5500M 8 GB graphics card. The experiment was divided into two parts: macro-expression migration learning and micro-expression recognition and classification. A grid search method was used to optimize the values of hyperparameters, including batch size, initial learning rate, and a number of iterations, before the start of each part of the experiment in order to make the model converge quickly during training and to save time in tuning parameters. The model is evaluated using a subject-independent evaluation protocol by taking all samples from one subject in each iteration as the test set and the rest of the subjects as the training set. The objects in the test set will therefore be different from all the objects in the training set and so may also contain different feature distributions. This way of evaluating the generalization ability of the model is gradually becoming more mainstream in micro-expression recognition tasks. In order to make a fair comparison with other studies, we use the unweighted recall rate (UAR) and the unweighted F1 score (UF1) as evaluation metrics alongside the accuracy rate. These metrics allow the accuracy of model recognition and the ability to balance between different classes to be measured. Assume that **TP**, **FP**, and **FN** are the true positive, false positive, and false negative, respectively. The UAR is calculated by $UAR=\frac{1}{C}\overset{C}{\underset{c=1}{\sum }}\frac{TP_{c}}{N_{c}}$,where ***TP***_***c***_ and ***N***_***c***_ are the number of true positives and all samples in the ***c***-th class, respectively. The UF1 is computed as $UF1=\frac{1}{C}\overset{C}{\underset{i=c}{\sum }}\frac{2P_{c}\times R_{c}}{P_{c}+R_{c}}$, where $P_{c}=\frac{TP_{c}}{TP_{c}+FP_{c}}$ and $R_{c}=\frac{TP_{c}}{TP_{c}+FN_{i}}$ for the ***c***-th class.

In the first part of the experiment, an SGD optimizer with a momentum of 0.9 was determined by a grid search, with an initial learning rate of 0.01 and a batch size of 32. In the second part, an Adam optimizer was determined by the same grid search, with an initial learning rate of 0.007 and a batch size of 64. The convolutional and fully connected layers of the model were subjected to L2 regularization at a scale of 0.2 and dropout at a scale of 0.5 to avoid overfitting. To improve the speed of each part of the experiment, the Keras 2.2.4 library was utilized.

### Experimental Results and Discussion

In order to demonstrate whether the use of transfer learning and the use of SVM classifiers are effective in improving the performance of the model, ablation experiments are necessary. The model was therefore subjected to three sets of comparison experiments, of which the performance of the model in different situations can be seen in Table 2. When the models were trained directly on the micro-expression dataset without transfer learning training, there were varying degrees of performance degradation on both the composite and individual datasets. The largest degradation is in the SMIC dataset, which we believe is likely due to the fact that the SMIC dataset has less data than the remaining two datasets, resulting in an underfitting of the model. There was also a decline in performance of the composite and separate datasets when the model did not employ an SVM classifier. Although a number of studies have used Softmax as a classifier, for the micro-expression recognition task, SVM was better at classifying between small differences. To further illustrate the effectiveness of transfer learning and SVM classifiers in improving the models, Table 2 also includes different neural networks for comparison experiments, showing that the performance of MobileNetV2 (Sandler et al., 2018), ResNet18, and the ViT model DeiT (Touvron et al., 2021) without convolutional layers without transfer training is lower than that of the models with transfer learning. The same is true for the SVM classifier in the other models.

**Table 2:** Comparative effectiveness of ablation experiments.

**Approach**	**Composite dataset**		**CASME II**		**SMIC**		**SAMM**	
	**UAR**	**UF1**	**UAR**	**UF1**	**UAR**	**UF1**	**UAR**	**UF1**
MobileViT without transfer learning	0.6348	0.6524	0.6571	0.6452	0.6018	0.6215	0.6398	0.6187
MobileViT without SVM classifier	0.6002	0.6128	0.6102	0.6237	0.6075	0.5925	0.5977	0.5921
MobileViT	0.6981	0.7318	0.6997	0.7251	0.7356	0.7141	0.6781	0.7428
MobileNetV2 without transfer learning	0.5721	0.5488	0.5815	0.5913	0.6018	0.6002	0.5741	0.6021
MobileNetV2 without SVM classifier	0.5981	0.6003	0.5749	0.5820	0.5991	0.6008	0.5952	0.6101
MobileNetV2 (Sandler et al., 2018)	0.6425	0.6652	0.6328	0.6125	0.6368	0.6589	0.6236	0.6614
ResNet18 without transfer learning	0.6028	0.6211	0.6238	0.6191	0.6233	0.6391	0.5937	0.6195
ResNet18 without SVM classifier	0.6331	0.6558	0.6244	0.6335	0.6025	0.6347	0.6273	0.6589
ResNet18	0.6682	0.6715	0.6522	0.6428	0.6271	0.6542	0.6632	0.6743
DeIT without transfer learning*	0.6526	0.6625	0.6471	0.6332	0.6514	0.6682	0.6689	0.6472
DeIT without SVM classifier*	0.6549	0.6382	0.6711	0.6697	0.6794	0.6810	0.6502	0.6602
DeiT* (Touvron et al., 2021)	0.6879	0.6731	0.6814	0.6994	0.6881	0.6970	0.7052	0.7028

The * symbol indicates that the model is a non-convolutional model, so the weights of the full model are preserved in that part of the experiment. The rest of the model only preserves the weights of the convolutional layers.

**Figure 6:**
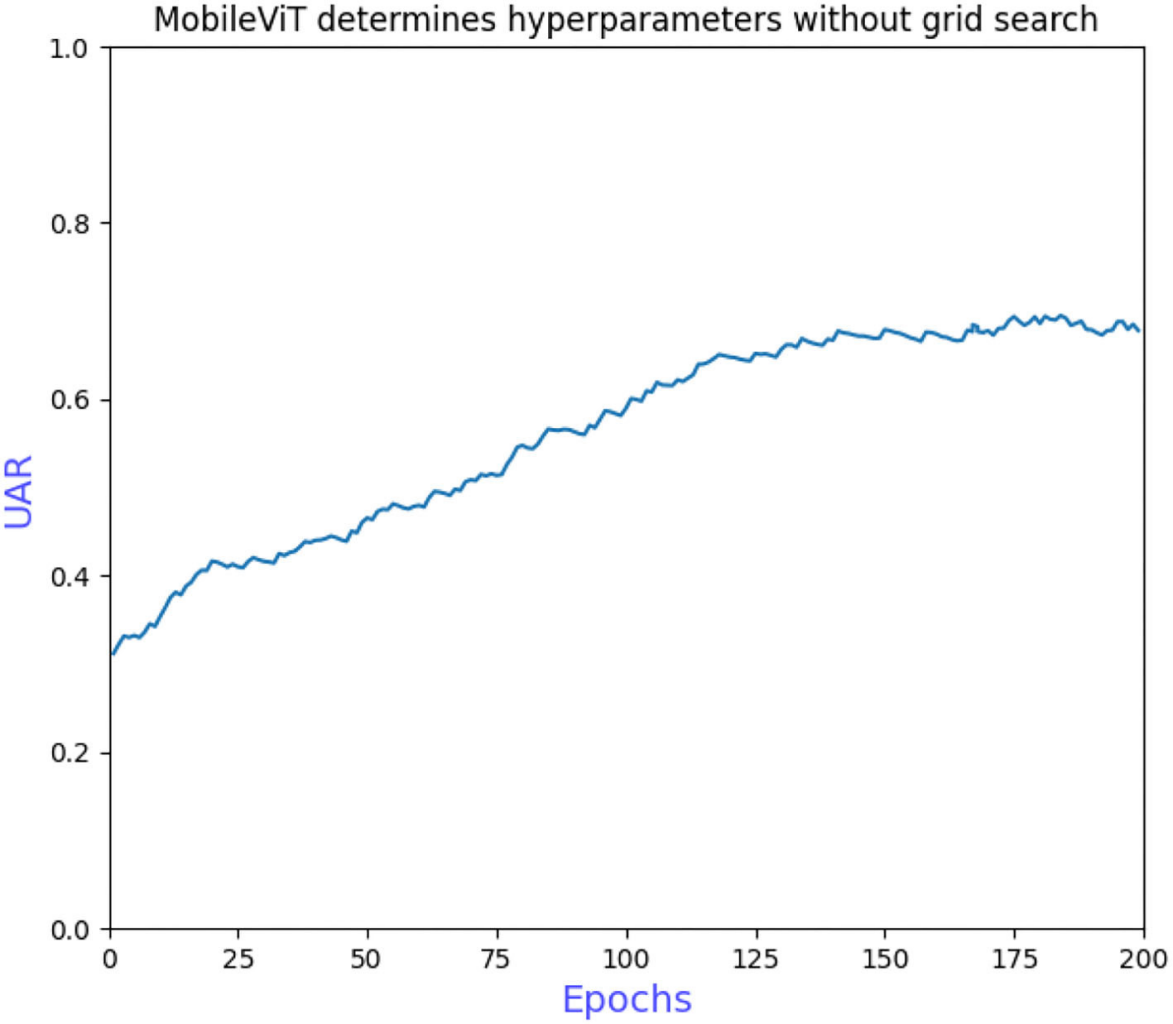
MobileViT determines hyperparameters without grid search.

**Figure 7:**
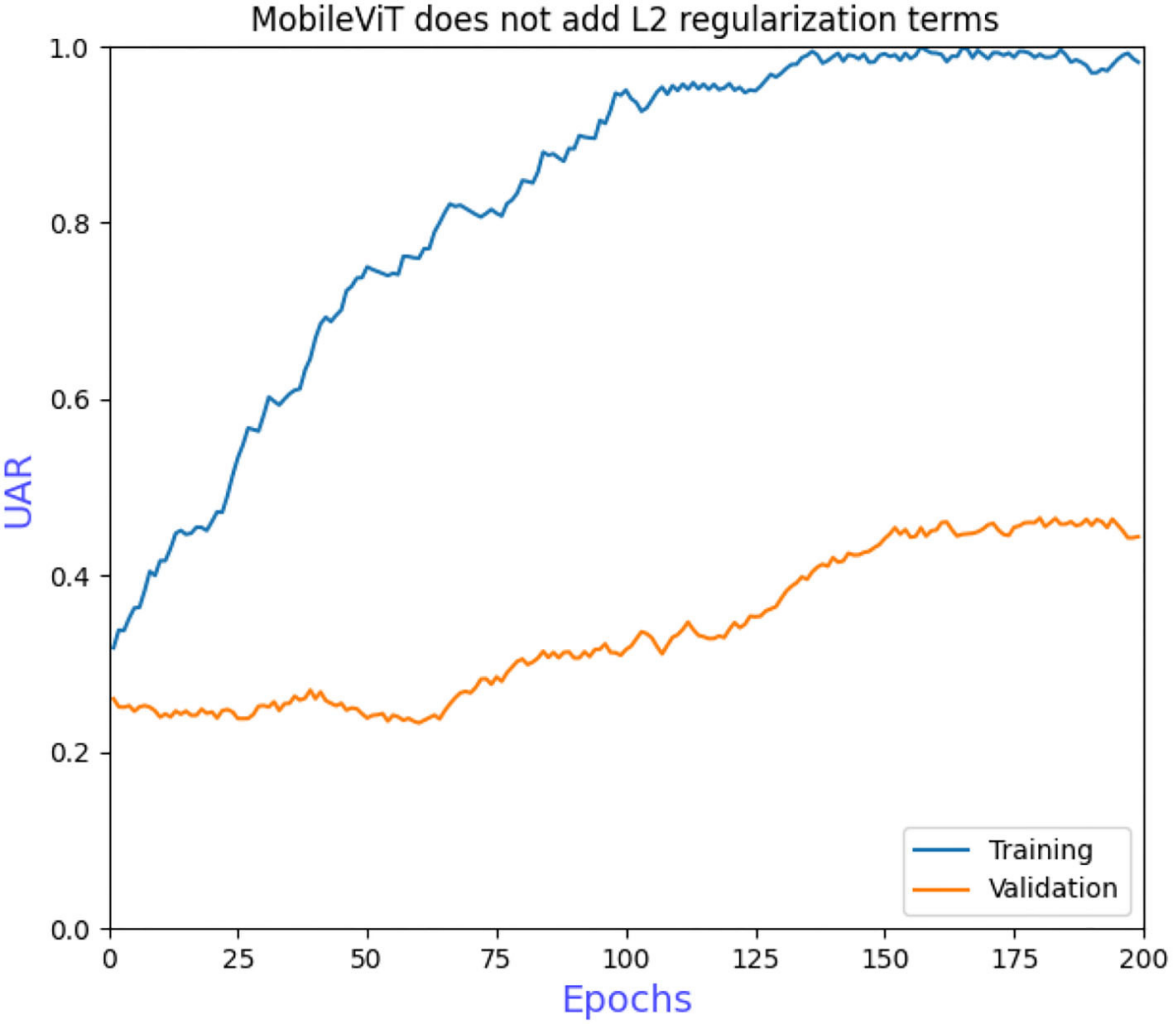
MobileViT does not add L2 regularization terms.

**Figure 8:**
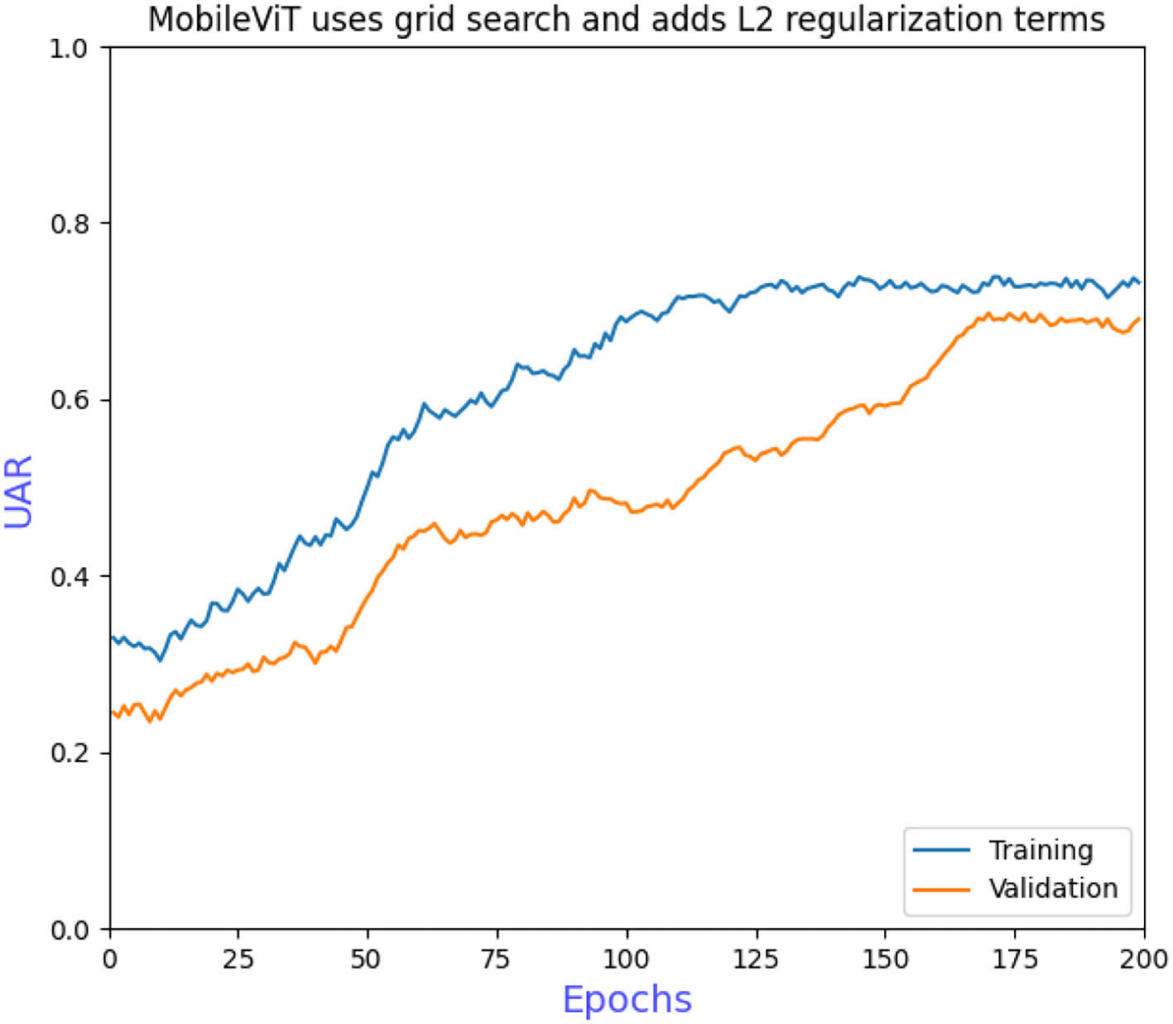
MobileViT uses grid search and adds L2 regularization terms.

The grid search to determine the hyperparameters also requires ablation experiments to prove the effectiveness of adding L2 regularization to the convolutional and fully connected layers of the model. Figure 6 shows that the model converges more slowly when trained without the grid search to determine the hyperparameters, requiring more iterations to achieve optimal performance. Figure 7 shows that overfitting occurs when L2 regularization is not added, that is, the training accuracy is close to 100% while the validation accuracy is only 43%, so overfitting measures are necessary on small datasets. Figure 8 shows that the training accuracy of the model after the grid search and the addition of regular terms has improved significantly compared to Figure 6 and does not show the overfitting in Figure 7.

Table 3 shows a comparison between the state-of-the-art approach and the proposed approach to demonstrate the performance of the model. First of all, the table contains some traditional methods, LBP-TOP (Pfister et al., 2011) is an appearance-based feature and was used as a baseline at MEGC2019, and the results have been published on their conference website. LBP-SIP (Wang et al., 2014), as an improvement of the LBP-TOP method, does show some improvement in performance. Whereas HOOF (Chaudhry et al., 2009) has been used as a baseline approach for optical flow features for a long time, the ROI-based optical flow feature approach proposed by MDMO (Liu et al., 2015) outperformed HOOF. MobileViT models achieve significantly better performance than several manual feature approaches. In fact, the deep models outperformed the manual feature extraction methods in most cases. Next, some of the deep networks were compared. ResNet18 and DenseNet121 achieved better results in a number of image classification problems as a baseline approach for deep networks, but the differences between the datasets used for training were more pronounced, the number of samples was more than adequate, and training on the micro-expression dataset would suffer inadequate training. The combination of CNN and LSTM was not used, and the number of micro-expression samples limited the performance of the model. The table also shows the most recent state-of-the-art approach RCN (Xia et al., 2020b), whose maximum value per dataset we took for comparison in our comparison experiments and whose proposed approach has good adaptability across datasets. The TSCNN is also one of the most advanced approaches, but MobileViT with migration learning performs significantly better. These comparisons also show that MobileViT blocks can effectively improve the accuracy of micro-expression recognition after extracting local and global information. MobileViT does not achieve exactly the same performance compared to these deep models, due to the fact that MobileViT is a lightweight network model that is superior in terms of training efficiency. Finally, MobileViT is similar to DeepViT (Zhou et al., 2021) and DeiT without convolutional layers and is closer in performance to the deep neural network models, but DeepViT and DeiT have 55 and 86 M parameters, making it difficult to run on platforms with poor hardware conditions.

**Table 3:** UAR and UF1 performance of different approaches under LOSO protocol on composite or individual datasets.

**Approach**	**Composite dataset**		**CASME II**		**SMIC**		**SAMM**	
	**UAR**	**UF1**	**UAR**	**UF1**	**UAR**	**UF1**	**UAR**	**UF1**
LBP-TOP (Pfister et al., 2011)	0.5785	0.5882	0.5429	0.5026	0.5280	0.2000	0.4102	0.3954
LBP-SIP (Wang et al., 2014)	0.4681	0.4829	0.5281	0.5369	0.5142	0.4452	0.4169	0.4412
HOOF (Chaudhry et al., 2009)	0.5814	0.5982	0.5782	0.5874	0.5696	0.5574	0.5877	0.5639
MDMO (Liu et al., 2015)	0.5125	0.5635	0.5382	0.5492	0.4812	0.4926	0.5108	0.5021
ResNet18	0.6682	0.6715	0.6522	0.6428	0.6271	0.6542	0.6632	0.6743
CNN-LSTM (Wang et al., 2018)	0.3942	0.3852	0.4125	0.4113	0.4276	0.4150	0.3086	0.3020
DenseNet121	0.3414	0.4253	0.3334	0.4604	0.3518	0.2909	0.3374	0.5645
RCN-Best (Xia et al., 2020b)	**0.7190**	**0.7466**	0.6600	0.6584	**0.8131**	**0.8653**	0.6771	**0.7647**
TSCNN (Song et al., 2019)	0.5849	0.5923	0.6009	0.6124	0.5924	0.5839	0.6103	0.6083
MobileNetV2 (Sandler et al., 2018)	0.6425	0.6652	0.6328	0.6125	0.6368	0.6589	0.6236	0.6614
DeepViT (Zhou et al., 2021)	**0.7025**	0.7158	**0.7001**	0.6982	0.7152	0.7369	**0.7114**	0.6928
DeiT (Touvron et al., 2021)	0.6879	0.6731	0.6814	**0.6994**	0.6881	0.6970	**0.7052**	0.7028
**MobileViT (Ours)**	0.6981	**0.7318**	**0.6997**	**0.7251**	**0.7356**	**0.7141**	0.6781	**0.7428**

*The bold values indicate the highest values under the particular metrics*.

**Figure 9:**
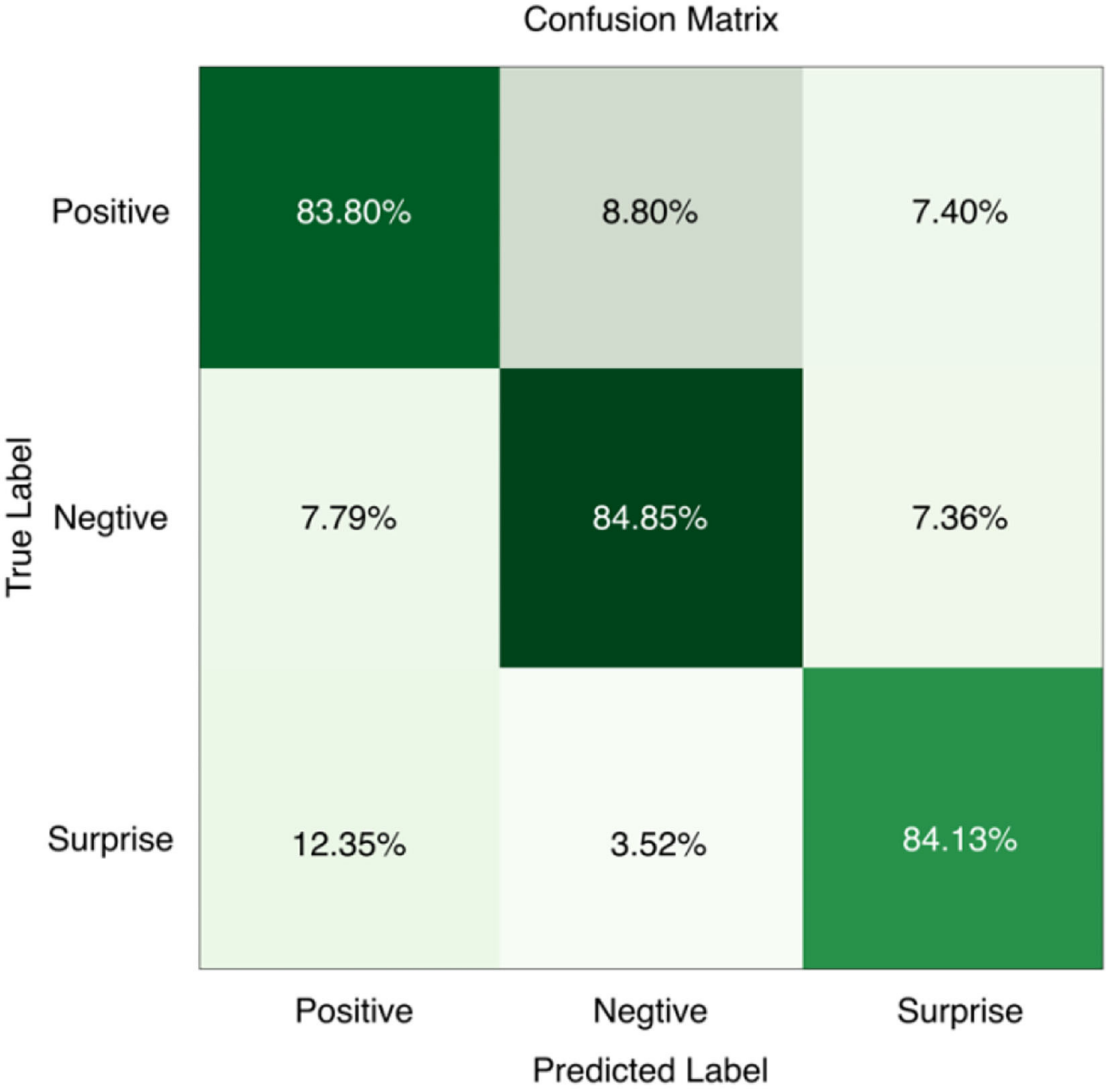
Confusion matrix of MobileViT training results on the composite micro-expression dataset.

In order to analyze in more detail the difference between the three types of micro-expression recognition results and the accuracy of different emotion categories, the confusion matrix was calculated, as shown in Figure 9. From the figure, it can be seen that negative emotions have the highest accuracy in the three emotion classification experiments. This is because the negative category has the largest number of emotion samples, and it is easier for the neural network to grasp its features during the training process. The positive and surprise emotions have lower recognition rates. The surprise class emotions have the lowest number, so it is difficult to obtain accurate features from a small amount of data.

In terms of recognition speed, the average time taken to recognize an expression by different models in the same experimental environment and equipment is compared in Table 4. The methods compared in the table are all baseline methods as many of the models do not provide the relevant source code, and it is difficult to reproduce their network structures. Compared to the lightweight MobileNetV3 model without the ViT structure, MobileViT takes slightly more time and has a significant advantage over ResNet18. However, compared to the model without the convolutional structure, the recognition efficiency is much improved and is slightly greater than the minimum duration of micro-expressions (30 ms), allowing facial micro-expressions to be captured in a timely and effective manner.

**Table 4:** Comparing the time to identify individual sentiment samples by different network models.

**Model**	**Time**
MobileNetV2	31.8 ms
DeiT	53.7 ms
ResNet18	82.3 ms
**Proposed Approaches**	**35.4 ms**

The pre-processing part of the data is given great importance in the experiments, and the images in the macro-expression dataset need to be processed as close as possible to the micro-expression images. At the same time, to avoid the problem of the unbalanced number of sample categories, the same number of emotion category samples is randomly selected in each training iteration in the experiment to prevent the neural network model from classifying the samples to a certain emotion category more favorably. The merged dataset also improves the generalization ability of the model during the training process and has better stability when facing new samples. In fact, the size of the facial action region for micro-expressions is very limited. Therefore, directly extracting the feature vector of the face using the complete facial samples will contain more redundant information, which will reduce the expressiveness of the feature vector and thus affect the recognition accuracy.

## Conclusion and Future Work

This study is the first application of self-attentive mechanisms to the field of micro-expression recognition. A migration learning approach and an optimized MobileViT model are proposed. The convolutional layer in MobileViT is trained to be a facial expression feature extractor with an excellent performance by migration learning, and a grid search is used to find the value of the best hyperparameter. The L2 regularization term is added to the convolutional and fully connected layers of MobileViT in order to avoid overfitting. MobileViT satisfies both the lightweight nature of the model and the access to the global representation. The method does not become more complex than lightweight CNNs by incorporating the ViT structure but, at the same time, achieves better performance than the convolution-free ViT structure, extracting the global representation while ensuring the lightweight of the model and obtaining a performance close to that of the state-of-the-art MER methods. In future work, on the one hand, we will continue to try to improve the transformer model to make it real time and able to run micro-expression recognition applications well in embedded devices. On the other hand, we will try to simulate the acquisition environment in different situations by adding perturbations to the micro-expression samples or by adjusting different parameters of the images and, thus, adjust the model to adapt to the stability of recognition in different environments and improve the robustness of the model.

## Data Availability Statement

The original contributions presented in the study are included in the article/supplementary materials, further inquiries can be directed to the corresponding author/s.

## Author Contributions

YangL and XY finished the main content of the manuscript. HZ and ZH prepared the data and carried out experiments. YanjL and YanzL finished all the drawings in the manuscript. All authors contributed to the article and approved the submitted version.

## Funding

This research was funded by National Natural Science Fund Youth Fund Project of China grant number No. 61403222, Heilongjiang Provincial Department of Education grant number No. 135309466.

## Conflict of Interest

The authors declare that the research was conducted in the absence of any commercial or financial relationships that could be construed as a potential conflict of interest.

## Publisher's Note

All claims expressed in this article are solely those of the authors and do not necessarily represent those of their affiliated organizations, or those of the publisher, the editors and the reviewers. Any product that may be evaluated in this article, or claim that may be made by its manufacturer, is not guaranteed or endorsed by the publisher.
